# Optical and UV Shielding Properties of Inorganic Nanoparticles Embedded in Polymethyl Methacrylate Nanocomposite Freestanding Films

**DOI:** 10.3390/polym16081048

**Published:** 2024-04-10

**Authors:** Kathalingam Adaikalam, Dhanasekaran Vikraman, Du-Hee Lee, Yoon-A Cho, Hyun-Seok Kim

**Affiliations:** 1 Millimeter-Wave Innovation Technology Research Center, Dongguk University-Seoul, Seoul 04620, Republic of Korea; kathu@dongguk.edu; 2 Division of Electronics and Electrical Engineering, Dongguk University-Seoul, Seoul 04620, Republic of Korea; dhana86@dongguk.edu (D.V.); ldood47@gmail.com (D.-H.L.); yoona0408@gmail.com (Y.-A.C.)

**Keywords:** polymethyl methacrylate, free-standing films, flexible films, carbon nanotubes, carbon quantum dots, titanium dioxide, nanocomposites

## Abstract

Polymethyl methacrylate (PMMA) is an interesting polymer employed in various applications due to its outstanding properties. However, its electrical and mechanical properties can be further improved by incorporating nanoparticles, and in particular, PMMA nanocomposite with nanoparticles provides various multifunctional properties. This work reports PMMA nanocomposite preparation and structural and optical characterizations incorporating carbon nanotubes (CNTs), TiO_2_ nanoparticles, and carbon quantum dots (CQDs). CNT/PMMA, TiO_2_/PMMA, and CQD/PMMA nanocomposite freestanding films were prepared using a simple solution method. Various properties of the prepared composite films were analyzed using scanning electron microscopy, X-ray diffraction, photoluminescence, Fourier transform infrared, and UV–Vis and Raman spectroscopy. Optical parameters and photocatalytic dye degradation for the films are reported, focusing on the properties of the materials. The CNT/PMMA, TiO_2_/PMMA, and CQD/PMMA films achieved, respectively, good electrical conductivity, photodegradation, and fluorescence compared with other composite films.

## 1. Introduction

Inorganic nanoparticles incorporating organic or polymeric nanocomposites have attracted considerable research interest due to outstanding properties suitable for various novel applications, providing a new nanocomposite class. Nanocomposites of organic and inorganic nanoparticles provide novel electrical and optical field properties along with improved mechanical qualities [1]. Therefore, considerable efforts have been devoted to designing novel nanocomposite materials with efficient multipurpose applications, including nanostructures within polymer matrices that decrease transparency and increase refractive index, making them more suitable for light-emitting diodes (LEDs). Specific optical properties can be tailored depending on the applications, e.g., antireflective coatings, which are vital for many optical applications.

Different nanoparticle inclusion in polymer-based composites can produce multifunctional nanocomposites for different applications. Polymer properties can be tuned advantageously by selecting suitable nanoparticle inclusions. Incorporating metal oxide or metal nanoparticles into organic polymeric matrices creates new state-of-the-art composite materials, exhibiting improved mechanical, electrical, and optical properties compared with the individual components. Several recent studies have prepared specific multipurpose polymer and metal oxide nanoparticle composites with metaproperties suitable for solar cells, LEDs, photodetectors, UV shielding, supercapacitors, chemical and biosensors, etc. The incorporation of quantum structures into a polymer matrix can provide linear and non-linear optical properties. Organic polymer matrices, including inorganic nanostructures, can provide functionalized molecular structures with new and exciting enhanced performances and properties.

Polymethyl methacrylate (PMMA) is widely employed as the host matrix for polymer and inorganic nanocomposite preparation, and it has considerable optoelectronic applications due to its interesting properties. The composite optical properties can be relatively easily tailored for various applications. For example, the refractive index can be tailored to produce antireflective or invisible coatings by including metallic oxide nanoparticles in the polymer matrix. Metallic oxide nanoparticle inclusion significantly increases the refractive index due to scattering. This scattering also depends on nanoparticle size, providing a mechanism to modify the composite apparent color. Thus, selecting appropriate filler materials allows for altering various optical properties for specific applications. These materials are also attractive for environmental applications, such as photocatalytic contaminant removal and antimicrobial films. PMMA polymer-based nanocomposite thin films have gained considerable attention for high transparency, lightweight, low thermal expansion, improved flexibility and mechanical strength, etc. [2].

PMMA is an amorphous thermoplastic material commonly used to fabricate interesting MEMS and metamaterials [3,4], offering improved biodegradability, relatively simple processability, and enhanced electrical, thermal, optical, and mechanical properties. Application fields include solar cells, photodetectors, LEDs, UV shielding, optical lenses, chemical and biosensors, energy storage devices, etc. These polymer nanocomposites are extensively used in various optoelectronic devices and coatings, and PMMA-based composite’s optical properties can be tailored for specific applications by varying the associated nanofiller components. Incorporating nanoparticles in PMMA can also alter fundamental optical properties, i.e., absorption, refraction, etc. [2]. Thus, considerable research has become focused on preparing multipurpose nanocomposite materials using PMMA-based nanocomposites, tailoring optical, mechanical, and other desirable physiochemical properties using different nanoparticles. Property changes depend strongly on nanoparticle size and shape; hence, various nanoparticle shapes and sizes were selected for this study, including carbon nanotubes (CNTs), TiO_2_ nanoparticles (NPs), and carbon quantum dots (CQDs). These materials also have significant potential for environmental applications, such as removing photocatalytic contaminants and removing antimicrobial films.

Carbon nanotubes are generally considered the best material to produce highly attractive PMMA nanocomposites for different applications, converting PMMA into highly conducting flexible material with good electrical and mechanical properties. PMMA and CNT nanocomposite materials have recently gained attention for multifunctional applications [5]. Moreover, along with other electrical and mechanical advantages, this CNT-impregnated CNT/PMMA nanocomposite can provide good antimicrobial freestanding films that are useful for medical applications [6]. Similarly, metal oxide (MO) inclusion is commonly used to form improved efficiency nanocomposites for various applications. In particular, titanium dioxide (TiO_2_) can help improve electrical, optical, and mechanical properties. TiO_2_ is a low-toxicity, highly stable oxide material with high catalytic properties, and TiO_2_ films can provide self-cleaning coatings due to hydrophobicity and photocatalytic properties [7]. TiO_2_ has been largely explored as an attractive photocatalytic material; it has good photochemical stability, photoactivity, and tunable properties [8,9,10]. Moreover, it is suitable for photo-induced catalytic and antimicrobial activities and is applicable for photocatalytic degradation of contaminants, including microbial contaminants [11]. TiO_2_ hydrophobicity combined with contaminant removal has been employed for anti-fouling and self-cleaning coatings useful for paints, textiles, household utensils, and many industrial factories. The PMMA/TiO_2_ films provide self-cleaning surfaces, avoiding or mitigating fouling problems. Solar light irradiation helps TiO_2_ not only break down organic substances but also kill bacteria and viruses attached to its surface. Quantum dots (QDs) are also nanoparticles with diameters < 10 nm, providing promising nanomaterials for a wide array of applications. These properties are entirely different from bulk counterparts due to quantum confinement effects, and hence, QDs offer unique electronic, optical, chemical, and mechanical properties attractive for many technological applications [12]. Carbon quantum dots in particular show great potential for many applications, achieving lower toxicity and cost, with more robust biocompatible compared with other quantum dot types [13].

The main objective of the current work was to synthesize different nanoparticles incorporated in PMMA nanocomposite films and study their usability. Hence, three different nanoparticle types, CNTs, TiO_2_ NPs, and CQDs, were selected and prepared for incorporating nanoparticles in PMMA matrices, denoted as CNT/PMMA, TiO_2_/PMMA, and CQD/PMMA freestanding films, respectively. We studied their feasibility for various applications and provided a relatively simple and efficient mechanism to produce nanofiller-incorporated PMMA nanocomposites as freestanding films. This report provides relevant properties for the prepared nanocomposite freestanding films and outlines their performance in various applications, focusing on the composite films’ structural, morphological, and optical properties.

## 2. Experimental Details

All chemicals were analytical grade, purchased from Sigma-Aldrich (St. Louis, MO, USA), and used as purchased to prepare the various films, etc. PMMA (850,000 g/mol) was also purchased from Sigma-Aldrich. Commercial single-walled CNTs, TiO_2_ powder (Degussa, P25, Zurich, Switzerland), and CQDs were used to prepare CNT/PMMA, TiO_2_/PMMA, and CQD/PMMA films, respectively. The nanoparticles should be disbursed uniformly within the PMMA solution to ensure they uniformly reinforce the PMMA matrix and form well-distributed PMMA-nanoparticle composite films. However, the nanoparticles generally cannot be uniformly dispersed by direct mixing with PMMA solution and require tailored strategies to homogeneously disperse them through the PMMA matrix. Thus, we employed ethanol as a convenient means of achieving uniform dispersion in the PMMA nanocomposite films.

**Figure 1:**
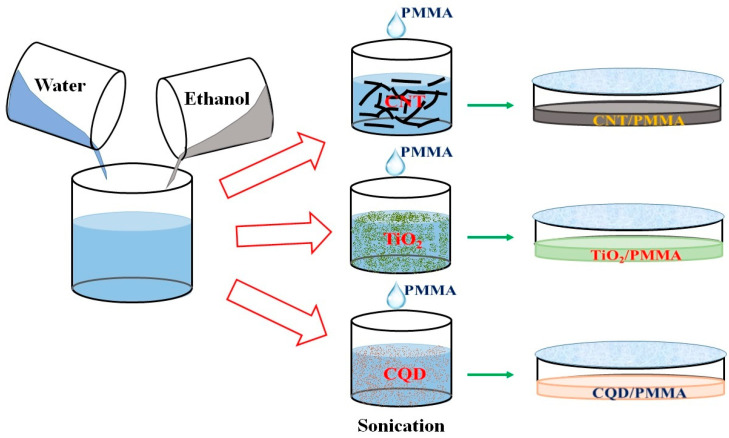
Typical experimental procedure to prepare CNT/PMMA, TiO_2_/PMMA, and CQD/PMMA nanocomposite freestanding films.

Figure 1 shows a typical experiment: three batches of 20 mL each of ethanol and deionized (DI) water were prepared by magnetic stirring in separate beakers. Then, 0.2 mg CNT, TiO_2_ powder, and CQD were mixed separately with prepared ethanol and DI water solution and sonicated for 1 h under ambient conditions. PMMA (10 mL) was then added dropwise into each solution separately under continuous stirring for 30 min, and the resultant solutions were sonicated for another 1 h. Finally, the nanoparticle solutions were poured into well-cleaned Petri dishes and left undisturbed for one week in ambient conditions. Formed films were removed from the Petri dishes and stored for further characterization and use.

Prepared film morphological structural and optical properties were characterized using field emission scanning electron microscopy (FE-SEM), X-ray diffraction (XRD), Fourier transform infrared spectroscopy (FTIR), photoluminescence (PL), and ultraviolet–visible (UV–Vis) spectroscopy techniques. Morphological and compositional properties were also studied using a scanning electron microscope (SEM, Philips, Model XL 30 SEM, Eindhoven, The Netherlands). XRD measurements were taken using a Bruker Discover D8 diffractometer (Billerica, MA, USA) with CuKα radiation and current–voltage (I–V) measurements using a Keithley 2611B source meter (Solon, OH, USA). Photo-dependent I–V characteristics were also measured for the films under white light illumination. The photocatalytic activity was studied by investigating methylene blue (MB) dye degradation under sunlight. Stock solutions for the photocatalytic study were prepared by dissolving 5 mg MB dye in 100 mL of DI water and then placing 10 mL stock solution into three Petri dishes. Small sections (2 × 2 cm) from the CNT/PMMA, TiO_2_/PMMA, and CQD/PMMA freestanding films were cut and immersed in the MB solution separately under various sunlight conditions, using different dye solutions for 30 and 60 min exposure times. The dye solution was subsequently investigated for UV–Vis absorption to estimate photocatalytic degradation efficiency. An electrochemical impedance spectroscopic (EIS) study was conducted using a conventional three-electrode configuration comprising Pt wire (counter electrode), Ag/AgCl (reference electrode), and prepared thin films (working electrode) with 0.5 M Na_2_SO_4_ as medium.

## 3. Results and Analysis

### 3.1. Morphological and Structural Studies

**Figure 2:**
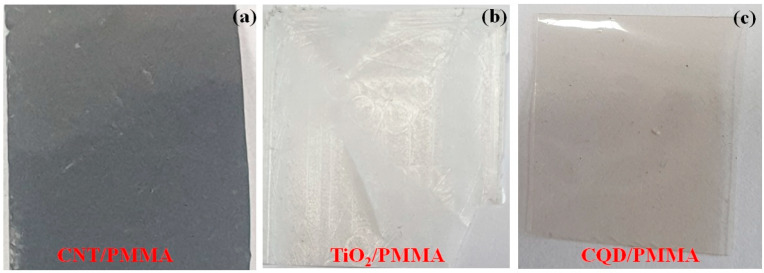
Photographic images for (**a**) CNT/PMMA, (**b**) TiO_2_/PMMA, and (**c**) CQD/PMMA composite freestanding films.

**Figure 3:**
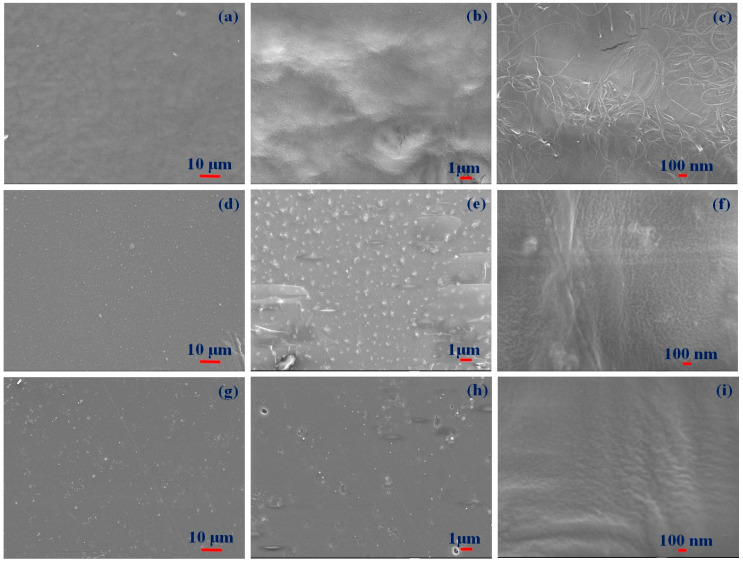
Field-effect scanning electron microscope (FE-SEM) images for (**a**–**c**) CNT/PMMA, (**d**–**f**) TiO_2_/PMMA, and (**g**–**i**) CQD/PMMA composite freestanding films at different magnifications.

**Figure 4:**
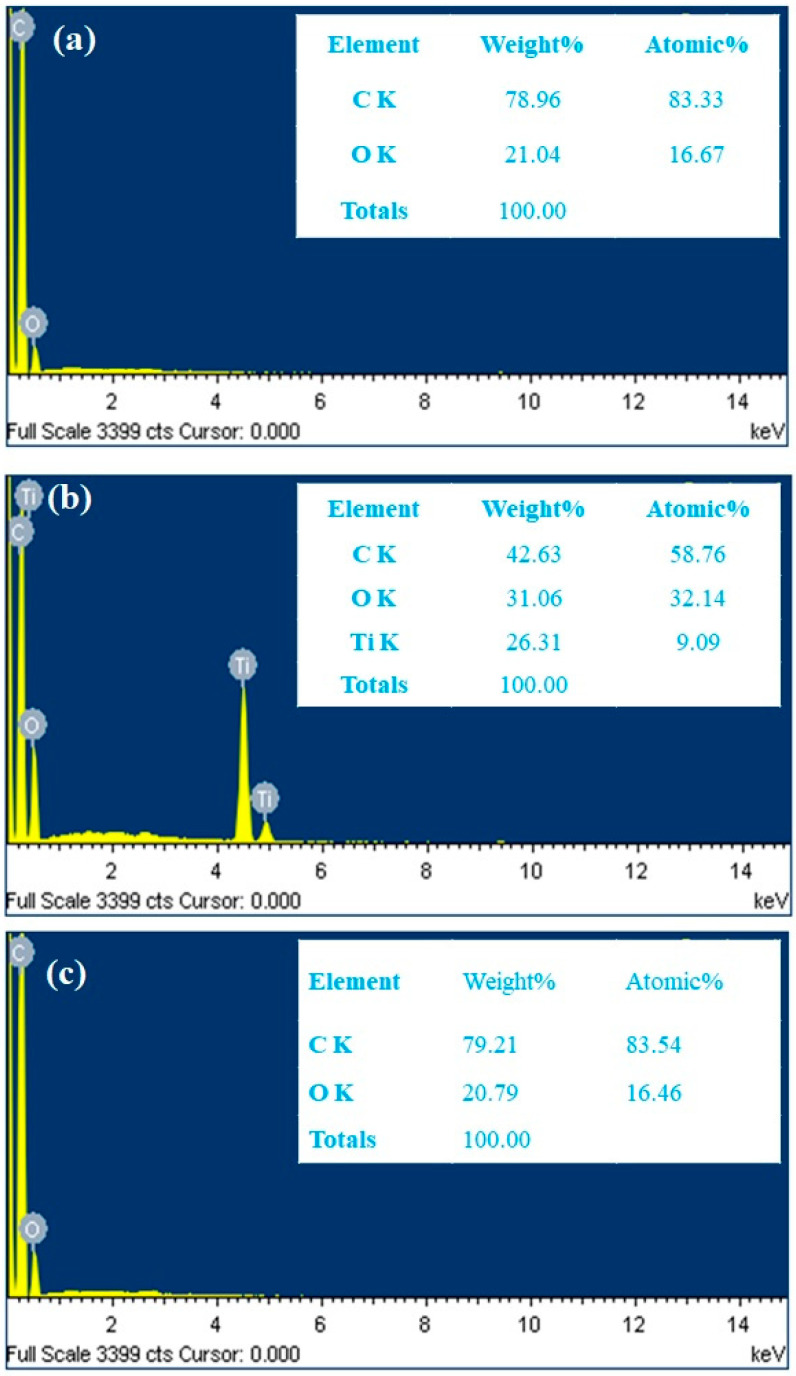
EDAX spectrum and elemental compositional for (**a**) CNT/PMMA, (**b**) TiO_2_/PMMA, and (**c**) CQD/PMMA composite films.

The prepared film’s surface morphological features were analyzed with SEM to estimate surface roughness, crystallinity, chemical composition, electrical conductivity, lubricity, and hydrophilicity. Figure 2 shows optical images indicating smooth surfaces with various transparencies and color appearances depending on filler material (CNT, TiO_2,_ or CQD). Figure 3 shows SEM images for the films at different magnifications, confirming smooth surfaces and indicating the filler materials within the PMMA matrix. CNT nanofillers impregnated within PMMA are clearly visible as randomly placed and interconnected fibers, whereas TiO_2_ nanoparticle composite films exhibit clustered and uniformly distributed nanoparticles, and CQD composite films also exhibit smooth surfaces, indicating clustered CQDs. Figure 4 shows elemental compositions for the PMMA nanocomposite films. CNT (Figure 4a) and CQD (Figure 4c) incorporated composite films that exhibit only C and O consistent with materials and ratio, whereas TiO_2_ incorporated exhibits C, O, and Ti as expected (Figure 4b). This also confirms CNT, TiO_2_, and CQD presence within the PMMA matrix in the prepared freestanding composite films.

**Figure 5:**
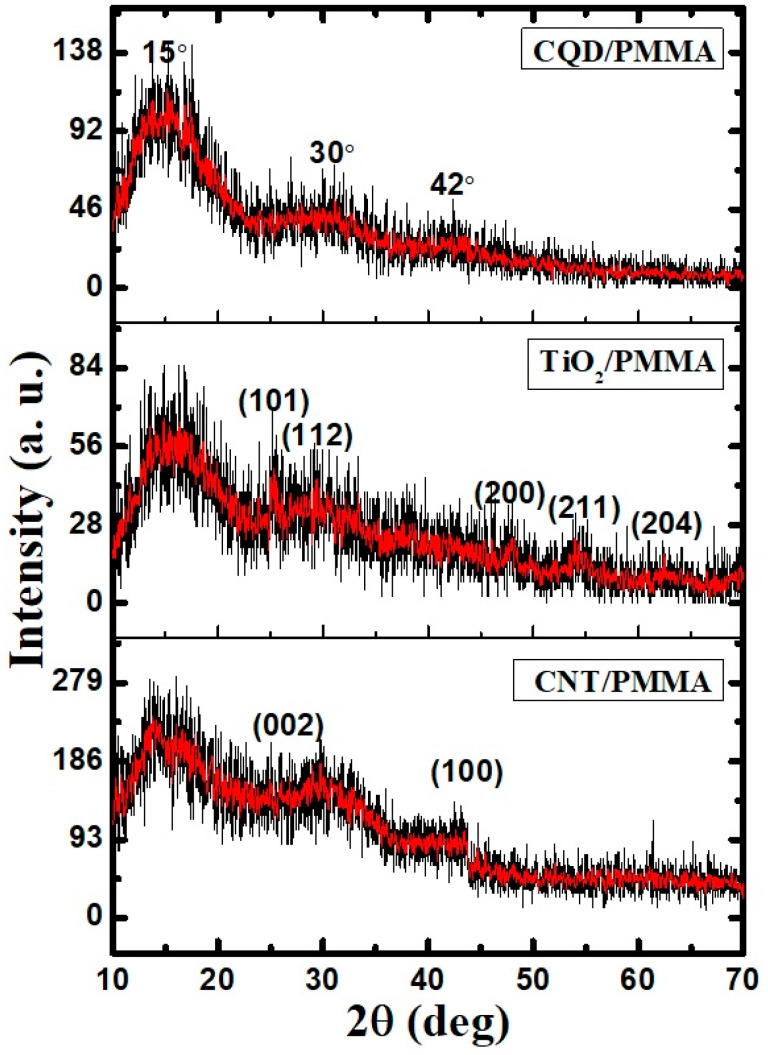
XRD patterns for CQD/PMMA, TiO_2_/PMMA, and CNT/PMMA composite freestanding films.

Figure 5 shows X-ray diffraction patterns for the prepared freestanding films. Wide peaks at 15°, 30°, and 42° indicate amorphous PMMA, and the large hump around 15° without sharp peaks confirms glassy amorphous PMMA for all the samples [14,15]. Sharp peaks at 26° and 43° for CNT/PMMA indicate (002) and (100) planes for CNT graphitic phase, respectively [16]. The large number of humps with less intensity indicates very low dimensional crystallite particle inclusions into the PMMA matrix. The TiO_2_ incorporated film exhibits several peaks, compared with CNT and CQD composite film, confirming TiO_2_ inclusion within the PMMA matrix. Peaks at 25°, 29°, 48°, 54°, and 62° correspond to (101), (112), (200), (211), and (204) anatase-structured TiO_2_ crystalline planes [17,18]. Thus, the prepared nanocomposites exhibit broadly similar amorphous nature.

### 3.2. Raman and FTIR Spectroscopic Studies

**Figure 6:**
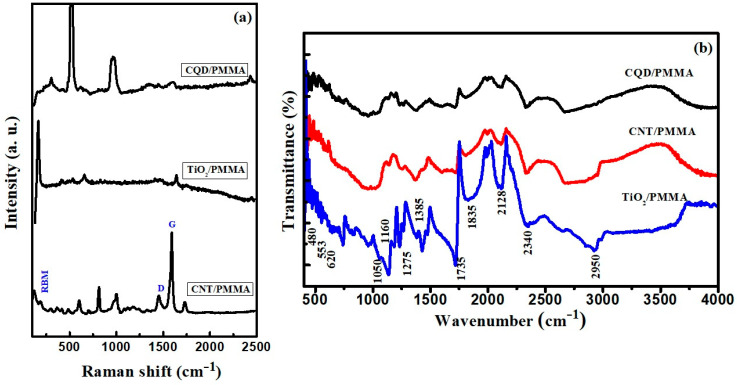
(**a**) Raman and (**b**) FTIR spectra for CNT/PMMA, TiO_2_/PMMA, and CQD/PMMA composite films.

Raman spectroscopy is a useful study for the structural analysis of carbon-based materials as it gives details of bonds and their related vibrations. Carbon-based nanomaterials show a number of overtones in Raman bands, and they are described based on graphene modes. Depending on the Raman peaks’ position, intensity, and shape, one can understand the material’s properties. Figure 6a shows the Raman spectrum of CNT/PMMA, TiO_2_/PMMA, and CQD/PMMA composite films. Characteristic Raman bands of CNTs such as D band (1450 cm^−1^), G-band (1585 cm^−1^), and radial breathing mode (RBM) peak around 200 cm^−1^ and are clearly seen on the CNT/PMMA, TiO_2_/PMMA, and CQD/PMMA films. Apart from in-plane structurally sensitive stronger bands, there are a number of weaker bands associated with shear and interlayer-activated defects [19]. Other peaks observed at 520, 315, 620, 815, 995, and 1725 cm^−1^ are associated with the PMMA matrix. Interference of plasmonic nanostructures with the PMMA matrix has resulted in multiple peaks with shifts [20].

Figure 6b shows Fourier transform infrared (FTIR) spectroscopy curves for CNT/PMMA, TiO_2_/PMMA, and CQD/PMMA nanocomposites. The spectra exhibit similar peaks and other features, such as PMMA vibrational bonds. However, exhibiting no sharp peaks confirms filler material interactions with PMMA molecules. Peaks 500–700 cm^−1^ were assigned to C-H bending, whereas peaks around 1000 cm^−1^ were assigned to -C-O-C- vibrations, and peaks 1160–1275 cm^−1^ to C-O bond stretching vibrations [1]. The bands around 1275–1500 cm^−1^ and 2700–3000 cm^−1^ are related to CH_2_ and CH_3_ vibrational modes. Peaks found at 1606 and 1740 cm^−1^ are due to C=C and C=O group stretching vibrations, respectively. Absorption bands around 2340 and 1810 cm^−1^ were attributed to CΞC and C-H stretching vibrations, respectively. Other unidentified peaks, e.g., at 1608 cm^−1^, may be due to adsorbed water bending and liberation modes [21]. Peaks observed at 450–800, 1630, and 3400 cm^−1^ are assigned to stretching vibrations of O-H, Ti-O, and Ti-O-Ti bonds due to surface adsorbed water and hydroxyl groups. The broad absorption band displayed in the 3400–3500 cm^−1^ range is due to Ti–OH vibration. Stretching vibrations of the C-O-C bond are also seen around 1000–1300 cm^−1^ [22,23], confirming the inclusion of TiO_2_ into the PMMA matrix. Almost all the peaks of different samples are the same and found like pure PMMA. This is because PMMA networks fully and continuously cover the nanostructures without disturbing its three-dimensional network. Moreover, the stretching vibrations of polymer group’s bands are in higher intensity.

### 3.3. Electrical Properties

**Figure 7:**
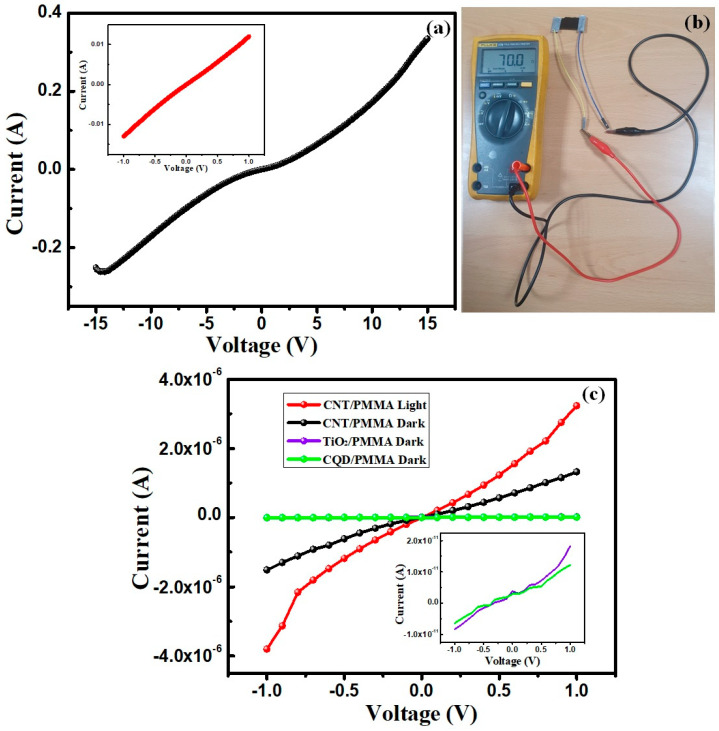
(**a**) Current–voltage curve (inset low voltage range), (**b**) observed resistance for the CNT/PMMA composite freestanding film, and (**c**) photocurrent–voltage plots of the films.

We calculated the electrical conductivity for the prepared composite films to help understand the electrical properties. TiO_2_/PMMA and CQD/PMMA films did not exhibit any conductivity, whereas CNT/PMMA composite films exhibited good conductivity ≈ at 12 S/cm, significantly higher than PMMA and other PMMA-based composite materials. This improved conductivity is due to the interlinked CNT fiber and its conductive nature. This interlinked continuity is absent for TiO_2_/PMMA and CQD/PMMA composite films since they form spherical nanoparticles into the PMMA matrix. Figure 7 shows the current–voltage response for the CNT/PMMA film over the −15 to 15 V range (with −1 to 1 voltage range in Figure 7a inset). The I–V linearity indicates low resistance ohmic behavior (Figure 7b). This improved CNT/PMMA composite freestanding film conductivity would be advantageous for catalytic and other applications as conductive electrodes replacing more expensive electrode options. The current–voltage response of the prepared films was also measured under different illumination conditions. Figure 7c displays the I–V plots of different freestanding films: CNT/PMMA, TiO_2_/PMMA, and CQD/PMMA. The figure shows that CNT/PMMA films showed photosensitive effects under white light illumination. In comparison, no photo response was observed for the TiO_2_/PMMA and CQD/PMMA films. They showed a very low current at dark, as shown in the inset of Figure 7c. Regarding room conditions, these films also showed very poor conductance, as stated earlier.

### 3.4. Optical Properties

**Figure 8:**
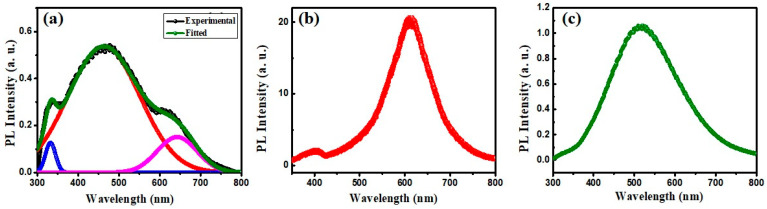
Photoluminescence spectra for (**a**) CNT/PMMA, (**b**) TiO_2_/PMMA, and (**c**) CQD/PMMA composite using excitation with 299 nm wavelength.

Figure 8 shows photoluminescence (PL) curves for the three prepared films to help analyze structure, defect states, and recombination properties. The CNT/PMMA composite film PL spectrum exhibits three emission peaks at UV (332 nm), green (470), and red (644) regions, with the strongest peak (Figure 8a), indicating CNT inclusion within the PMMA matrix [24]. The wide band observed in the 400 to 600 region centered at 470 nm represents the surface trap states of SWCNTs incorporated into the polymer matrix. The inclusion of CNT into the PMMA matrix forms a number of intermolecular hydrogen bonds between OH groups of PMMA and CNT, which can alter the optical properties of the composites, including refractive index [25]. The wide emission band at 600 nm for the TiO_2_/PMMA freestanding film (Figure 8b) indicates defect states produced by radiative excitons recombination in TiO_2_ [26]. The CQD/PMMA composite film PL emission spectrum (Figure 8c) exhibits a wide emission spectrum centered on 500 nm, suggesting CQD surface trap states [27]. The low emission intensity for the composite films compared with their individual counterpart nanoparticles indicates the PMMA matrix effect on recombination processes.

### 3.5. Electrochemical Impedance Analysis

**Figure 9:**
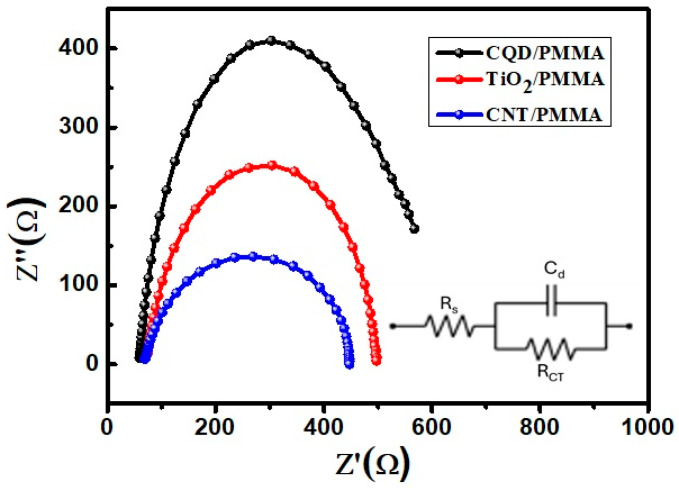
EIS Nyquist plots of CQD/PMMA, TiO_2_/PMMA, and CNT/PMMA composite films.

An EIS analysis of SWCNT/PMMA, TiO_2_/PMMA, and CQD/PMMA freestanding films was recorded at 10 mV applied voltage between 100 kHz and 0.01 Hz. Figure 9 displays the Nyquist plots obtained for the films showing semicircles in the low-frequency range. This semicircle nature without any straight-line portion indicates that they are not good for electrochemical applications. However, the semicircle nature of the plots indicates that the films have low charge-transfer resistance. Compared to CQD/PMMA and TiO_2_/PMMA films, the CNT/PMMA films have produced low-diameter semicircles, justifying their conducting nature with low resistance. These observed results indicate the usefulness of CNT/PMMA freestanding film for catalytic applications.

#### 3.5.1. UV–Vis Absorbance and Transmittance

**Figure 10:**
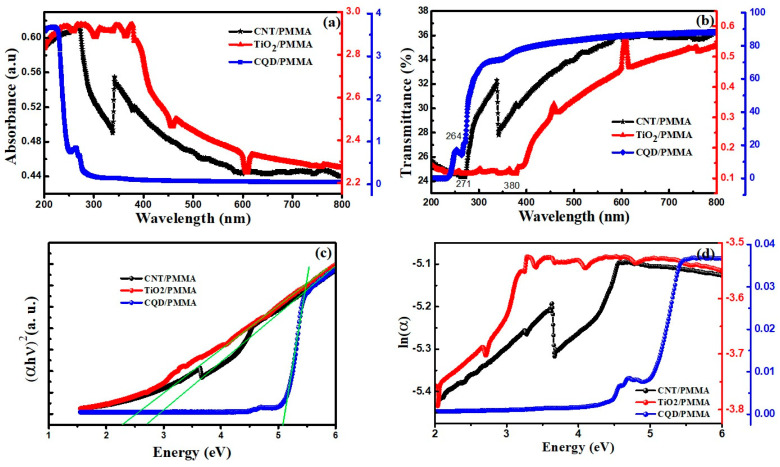
CNT/PMMA, TiO_2_/PMMA, and CQD/PMMA film (**a**) UV–Vis absorption, (**b**) transmittance spectra, (**c**) Tauc plot, and (**d**) Urbach energy.

Figure 10 shows absorbance and transmittance UV–Vis spectra for the three composite films at room temperature. Bandgap, refractive index, dielectric constants, etc., exhibit two distinct behaviors depending on the optical energy wavelength. Low wavelength absorbance is high, with less transmittance depending on the filler materials, whereas high wavelength region absorbance is reduced for all cases, confirming high transmittance. All the composites exhibit increased absorbance at low wavelengths, and the absorption edge is redshifted in the following order: CQD/PMMA, CNT/PMMA, and TiO_2_/PMMA, indicating reduced bandgap energies (Figure 10a). This absorbance in the UV region is due to electron transitions among energy states. Compared to the CQD-incorporated polymer nanocomposite, the CNT and TiO_2_-incorporated composites have shown absorption in the visible region, and there is more absorption for the TiO_2_/PMMA nanocomposite. In comparison, the CQD showed absorption only in the UV region below 270 nm, which is higher than the other composites.

Figure 10b shows transmittance spectra for the composite films. The CQD/PMMA composite film achieved the highest transmittance (≈90%) compared with other composite films. Thus, CQD-incorporated films achieved high transmittance across the whole visible region, reducing slowly with reducing frequency through the UV, decreasing markedly beyond 280 nm. The other composites also exhibit increased transmittance across the visible region, attaining maxims within the infrared region. The tip-like absorption edge for CQD and CNT incorporated composite films at 265 and 340 nm, respectively, can be attributed to graphite-dependent exciton-shifted Van Hove singularity effects [28]. A similar type of tip absorption also occurs for TiO_2_/PMMA at 455–607 nm, indicating PMMA molecule interactions with TiO_2_. The TiO_2_/PMMA film exhibited enhanced absorption up to 350 nm compared with CQD and CNT due to the TiO_2_ nanoparticle’s inherent photo-reactive electronic states.

CNT/PMMA composited has shown a sudden increase in absorbance at 338 nm with continued decrease for an increase in wavelength. It is due to the interaction of quantum structure with its adsorbed materials. The inclusion of nanoparticles into PMMA can increase the refractive index of the composite [22]. This increased refractive index can also increase the absorption coefficient of the composite material, causing an increased probability of fluorescence and producing an abrupt change in the absorbance of the composite, causing absorption variations [25]. It is a Fano resonance-like feature obtained due to quantum structure-induced plasmonic vibrations [29]. This Fano resonance is the resonance vibration produced by the interference of localized and continuum states of the nanocomposite material. A number of reports have stated a similar effect in the nanoparticles, including PMMA nanocomposites [30,31]. Similarly, the zigzag nature of absorption in the TiO_2_/PMMA composite is also a quantum effect-induced phenomenon. The incorporation of nanostructured materials into a PMMA matrix can induce quantum-induced properties. Moreover, the water trapped in the pores of nanostructures by experimental procedures can show an altered refractive index, causing a change in absorption [30]. Graphene, like layered materials or quantum dots, can exhibit higher absorption due to plasmonic and excitonic coupled transitions [32]. Depending upon the nature of adsorbed functional groups, hyperchromic or quenching effects may be produced.

Optical bandgaps for the composite materials can be extracted using the absorption spectrum since absorption for a material depends on electron excitation from the valence to the conduction band [26]. Thus, the absorption edge determines the material bandgap. The absorption edge increases for increasing wavelength (redshift) in the following order: CQD, CNT, and TiO_2_, indicating reducing bandgap in the same order. Bandgap energy (*E_g_*) for the composite films was estimated from the Tauc plot (Figure 10c),

(1) $$(\alpha h\vartheta )=B{\left(h\vartheta -E_{g}\right)}^{\gamma }$$

where *B* is a constant, *hν* is incident energy, α is the absorption coefficient, and *γ* = ½ for direct transition and 2 for indirect transition.

Thus, nanocomposite estimated bandgap for direct transition = 2.8, 2.28, and 5.07 eV for CNT/PMMA, TiO_2_/PMMA, and CQD/PMMA films, respectively. CQD/PMMA achieved a bandgap almost equal to pristine PMMA, whereas TiO_2_/PMMA and CNT/PMMA showed smaller bandgaps in the PMMA matrix and nanofiller particles [15]. This reduction indicates localized modifications and defect states due to nanofiller interactions with PMMA. This redshift is attributed to charge transfer between nanofillers within the PMMA matrix [1]. The in-gap surface energy states created due to oxygen vacancies present in oxide materials cause absorption below the bandgap absorbance, producing spikes in the spectrum. The absorption band at 275 nm is attributed to the *n*-π* transition for the C=O group in the CNT/PMMA composite [28]. Low-intensity TiO_2_/PMMA film transmission is due to PMMA matrix molecule-induced recombination inhibition [33]. Increased absorbance in the UV–Vis region suggests that nanoparticle loading can boost UV absorbance suitable for UV shielding applications.

Nanoparticle bandgaps for incorporated PMMA nanocomposite films are lower than their respective pure nanoparticles. TiO_2_/PMMA composites exhibit the lowest bandgap (2.28 eV) compared with 3.8 eV for TiO_2_ alone. This reduction in the composite is due to extending the absorption tail deep into the forbidden gap due to the localized states produced by functionalization-induced defect states of the composite materials. These localized defect states trap excited electrons, causing interstate levels to form a tail in the absorption spectrum. This tail is called the Urbach tail with associated Urbach energy *Eu*, which can be estimated from the Urbach equation,

(2) $$\alpha =\alpha_{0}exp\left(\frac{E}{E_{u}}\right)$$

where α is the absorption coefficient, *E* is the photon energy, and *Eu* is the Urbach energy [34].

Figure 10d shows calculated *Eu* between ln (α) and *E* for all the composites. Urbach energy = 7.25, 8.46, and 0.25 for CNT/PMMA, TiO_2_/PMMA, and CQD/PMMA composite films, respectively, calculated from the slope of the linear portion of the curves below the optical bandgap. These high *Eu* values are due to organic PMMA and water molecule interaction with CNT and TiO_2_ nanoparticle surfaces [35]. CQD/PMMA exhibits less *Eu* compared to CNT and TiO_2_ composites, indicating a less defect-induced change in *Eu* due to side effects. It is also consistent with the CQD/PMMA composite bandgap being equal to pure PMMA.

#### 3.5.2. Refractive Index

The refractive index and extinction coefficients help define the optical properties of thin films. The complex refractive index (*N*) for composite films can be expressed as the sum of refractive index (*n*) and extinction coefficient (*k*), i.e., *N* = *n* + *ik* [36]. However, an accurate estimation of *n* for these polymer-blended nanoparticles is not possible since there is a change in crystallinity, band structure, and absorption rate. Nevertheless, *n* and *k* can be calculated as [37]

(3) $$n=\left(\frac{1+R}{1-R}\right)+\sqrt{\left(\frac{4R}{{\left(1-R\right)}^{2}}\right)-k^{2}},$$

(4) $$k=\frac{\alpha \lambda }{4\pi },$$

and

(5) $$\alpha =\frac{2.303\times A}{l},$$

where *R* is the reflectance, *α* is the absorption coefficient, *A* is the absorbance, and *l* is the film thickness.

Organic polymeric materials generally exhibit smaller refractive indices; however, blending with inorganic or oxide nanoparticles exhibits higher refractive indices, which are more suitable for photonic devices. In particular, combining QDs’ quantum and polymeric materials can improve the hybrid material’s optical activity due to quantum electrooptical effects [38]. Nanostructure surface functionalization can also modify physical and optical properties, significantly altering the refractive index largely [39].

**Figure 11:**
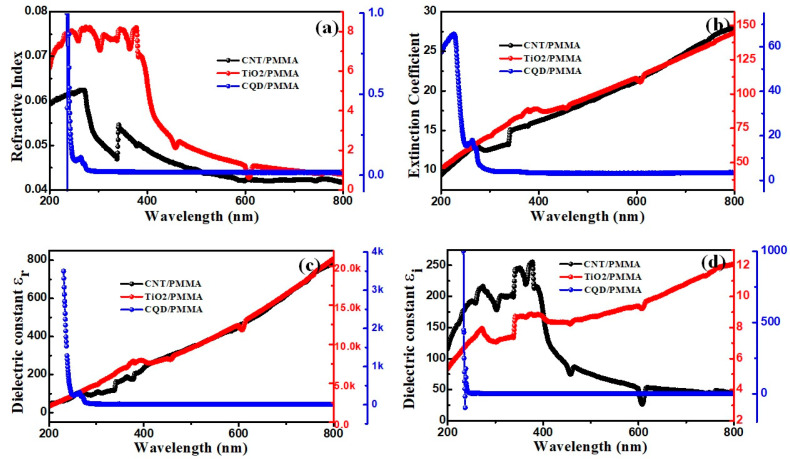
CNT/PMMA, TiO_2_/PMMA, and CQD/PMMA films’ (**a**) refractive index, (**b**) extinction coefficient, (**c**) dielectric constant real part, and (**d**) dielectric constant imaginary part with respect to optical wavelength.

Figure 11a shows the refractive index curves for the three composite films with respect to light wavelength. The CNT/PMMA and CQD/PMMA films exhibit a lower refractive index than TiO_2_/PMMA. The TiO_2_/PMMA film achieves *n* = 7.5 for 250–400 nm, reducing to *n* ≈ 1.2 at about 600 nm. In contrast, CQD/PMMA film achieves *n* = 1.2 at 230 nm, reducing to *n* = 0.2 at 300 nm and remaining constant at 300–800 nm. The CNT/PMMA film achieves very low *n* ≈ 0.06–0.04 for 250–800 nm. This decreased *n* at higher wavelengths indicates increased transmission at higher wavelengths. Figure 11b shows the extinction coefficient (*k*) for the composite films with respect to wavelength. CQD/PMMA composite film exhibits sharply decreasing *k* for 280–350 nm, then remains relatively constant at very small *k*, indicating little or no light loss in this range. In contrast, CNT and TiO_2_ composites exhibit increasing *k* with increasing wavelength, indicating large light dissipation due to nanoparticle absorption and scattering [40].

#### 3.5.3. Dielectric Constant

Optical energy absorption produces electronic polarization due to light interactions with the material [41]. This optical energy-dependent electronic polarizability and permittivity for composite films can be identified using the dielectric constant. Electrical insulation, related to the forbidden energy gap and density of states, can also be identified using the dielectric constant.

(*ω*) = *ε*_r_(*ω*) + *iε*_i_(*ω*), (6)

where *ε*_r_ and *ε*_i_ are the real and imaginary parts of the dielectric constant, respectively, and can be obtained from *n* and *k* as [37]

(7) $${Ԑ}_{r}=n^{2}-k^{2},\;{Ԑ}_{i}=2nk;$$

where Ԑ_r_ and Ԑ_i_ are the real and imaginary parts of the dielectric constant, respectively; the real part of the dielectric constant explains the reduced speed of light in the material, whereas the imaginary part indicates light energy absorption by the material due to dipole motion.

Figure 11c,d shows calculated real and imaginary parts for the film’s dielectric constants with respect to wavelength. The real part is always higher than the respective imaginary part. CNT/PMMA and TiO_2_/PMMA exhibit increasing *ε*_r_ with increasing wavelength, whereas CQD/PMMA exhibits decreasing *ε*_r_ for increasing wavelength. Thus, CQD/PMMA exhibits markedly different behavior from both CNT/PMMA and TiO_2_/PMMA, consistent with its relatively transparent for 300–800 nm.

#### 3.5.4. Fluorescence Spectroscopy

**Figure 12:**
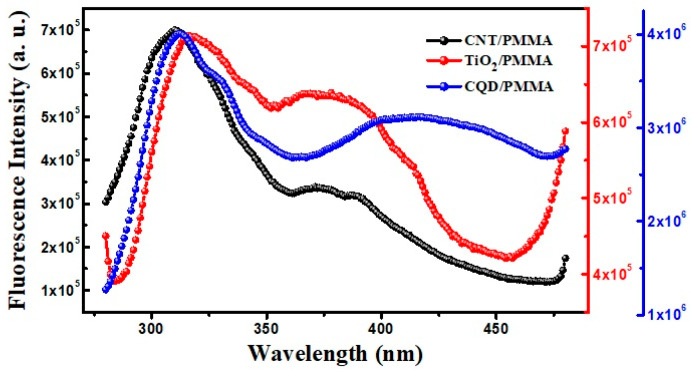
Fluorescence intensity for CNT/PMMA, TiO_2_/PMMA, and CQD/PMMA films with respect to the excitation wavelength.

Figure 12 shows fluorescent emission spectra for the PMMA-based composite films after excitation with UV light (360 nm). PMMA is an outstanding matrix material for oxide and other nanofillers; their luminescent spectra are not due to simple matrix and filler material superposition but depend on many interaction factors among the composite materials [42]. Nanoparticles smaller than organic molecular levels impregnated with polymer chains can exhibit enhanced optical properties [43]. Including nanoparticles in the PMMA matrix contributes to various functional groups and emission sites, resulting in multicolor fluorescence effects depending on excitation wavelengths. When the exciton pairs are confined within a space smaller than a Bohr radius, the band gap is widened, causing blue fluorescence emission [44]. Thus, fluorescence is an effective method to analyze the effects of material interaction on a composite.

All the composite films exhibit fluorescence emission from 300 to 350 nm centered around 320 nm with a slight shift towards higher wavelengths depending on the nanofiller materials (see Figure 12). No fluorescence emission was obtained at wavelengths above 500 nm. TiO_2_/PMMA and CQD/PMMA films exhibit additional wide emission bands at about 370 and 425 nm, respectively, compared with CNT/PMMA. CQD/PMMA exhibits wide fluorescence from 350 to 475 nm, indicating that its fluorescence is in the visible range due to quantum confinement [43]. CQD/PMMA film luminescence in the visible range can be exploited to enhance external quantum efficiency in photovoltaic cells by converting UV into visible light [45]. Carbon dots are the best fluorescent nanomaterial type; nanocomposite films with polymers can have interesting applications in optical fields due to this fluorescence emission.

#### 3.5.5. UV Shielding

Polymer-based nanocomposite films can be used to protect against UV and IR rays since they highly absorb UV and transmit visible light [46]. Organic compounds are generally degraded relatively easily by UV rays, reducing their UV-absorbing efficiency. However, including inorganic ingredients TiO_2_, CNT, and CQD can improve biocompatibility, reducing polymer degradation [47]. Although opaqueness and photocatalytic effects are improved, the effect depends on filler material size and other properties, deviating from optical properties [46,48]. TiO_2_-incorporated polymer nanocomposite films can act as excellent anti-fouling and self-cleaning coatings. UV light-induced reactive oxygen species help kill microorganisms and degrade organic foulants on surfaces [7]. This enhanced UV shielding from nanoparticle-incorporated PMMA films makes them uniquely suitable for optical devices, such as flexible and transparent screens, which need UV protection [49]. These nanoparticle-incorporated polymer composites could also be used as a photocatalytic agent to remove contaminants from water. The UV shielding property of TiO_2_/PMMA freestanding film was also experimentally examined as it showed higher absorption in the UV range. TiO_2_ commercial nanopowder was dispersed into MB dye solution and covered the solution-containing beaker with TiO_2_/PMMA films on the top and sides, as shown in Figure S1. Absorbance spectrum of the MB dyes with and without cover of the TiO_2_/PMMA film was obtained, and these spectra are also shown in Figure S1. As shown in the figure, the UV shielded dye shows almost equal absorbance to pure MB dye, whereas UV exposed MB dye showed low absorbance, indicating its UV-induced degraded nature. It confirms the UV shielding property of the TiO_2_/PMMA film.

### 3.6. Photocatalytic Effects

**Figure 13:**
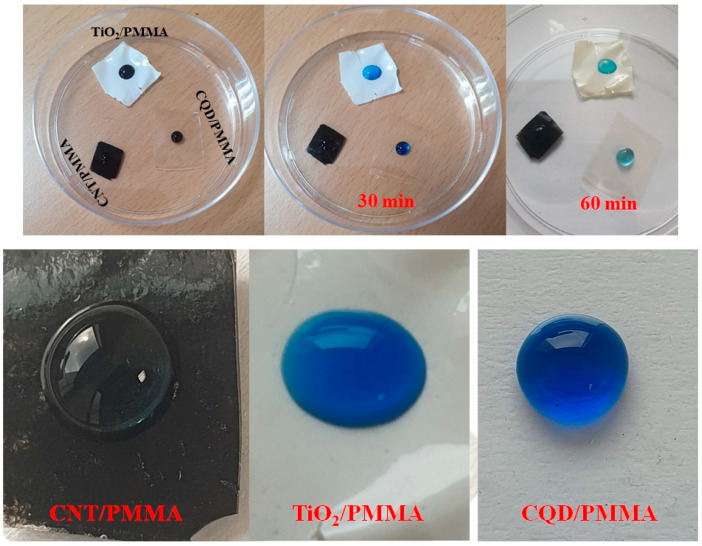
Methylene blue (MB) solution drops on CNT/PMMA, TiO_2_/PMMA, and CQD/PMMA films and corresponding color change under sunlight exposure for different intervals; magnified MB drops displaying composite film hydrophobicity.

To assess photocatalytic effects for the prepared composite films, methylene blue (MB) dye decolorization was assessed by measuring MB dye solution absorption after sunlight irradiation for different immersion times in the dye solution. MB has good absorption in the visible region, allowing relatively simple photocatalytic studies. To check the dye degradability of the films, a small drop of MB dye was put on each nanocomposite film, as shown in Figure 13. All the films exhibited color change depending upon exposure time (30 or 60 min under direct sunlight). An interesting hydrophobicity phenomenon was also evident (see Figure 13). CQD/PMMA exhibited more hydrophobicity than the other two films, retaining an almost perfect spherical droplet of MB.

To estimate photocatalytic decolorization, the films were immersed separately in MB dye solution using Petri dishes and exposed to sunlight for 30 and 60 min. Color changes were dependent on exposure time, indicating decreasing dye concentration due to photocatalytic dye degradation. Degradation was estimated from the degraded dye solution UV–Vis absorption spectra and degradation efficiency was calculated as

(8) $$Photocatalytic\;efficiency\;\left(\%\right)=\frac{A_{0}-A_{t}}{A_{0}}\times 100$$

where *A*_0_ and *A_t_* are initial and post-irradiation MB dye absorbance, respectively.

**Figure 14:**
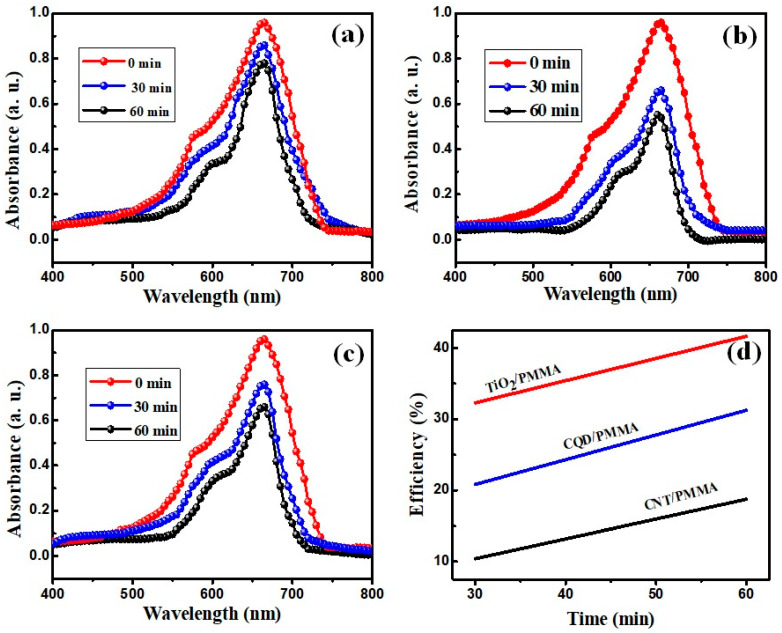
Methylene blue solution spectra after sunlight exposure for different intervals resting on composite films: (**a**) CNT/PMMA, (**b**) TiO_2_/PMMA, (**c**) CQD/PMMA and (**d**) their efficiency variation.

Figure 14 compares decolorization for the different films and exposure times, confirming decreased absorbance with increased exposure. Figure 14d shows that TiO_2_/PMMA achieved the highest degradation for MB dye, whereas CNT/PMMA composite exhibits very little effect. TiO_2_ is a good photocatalyst for photodegradation and, hence, achieved the highest MB degradation when present in the composite with PMMA [16]. Therefore, TiO_2_/PMMA films could be useful in removing hazardous dyes (and similar effects) for wastewater treatments. This freestanding nanocomposite film has produced poor efficiency compared to other nanocomposite powders, as shown in Table S1 and the review report presented by Idrees Khan et al. [50]. Comparing this freestanding film, powder catalysts can disperse efficiently into the dyes and react well, yielding high efficiency depending on materials and structural properties, whereas, in this film-based catalysts, only the surface is affected; it can be used as a self-cleaning adhesive.

The photodegradation process proceeds as follows: Hole and electron charges are excited to the nanoparticle valance and conduction bands due to photoexcitation. These excited electrons are transferred to the nanoparticle surfaces, where they react with dissolved oxygen and hydroxyl species, producing ionic radicals. The ionic radicals convert the pollutant dye molecules into H_2_O and CO_2_, degrading the dye contaminant [10]. The oxide and carbon nanoparticle-impregnated PMMA nanocomposites can not only remove organic substances adsorbed on their surface but can also kill bacteria and viruses via photo-induced reactive oxygen species [7,11]. Thus, these coatings can provide self-cleaning layers requiring no additional cleaning methods. The catalytic activity of TiO_2_ can be tuned by the action of adsorbed polymeric functional groups [51]. This polymeric association can reduce the bandgap of TiO_2_ and semiconducting metal oxides, which can increase electron/hole recombination rates, improving photocatalytic activity by increasing electron–hole separation under the action of light. Photocatalytic activity of TiO_2_ or any metal oxide photocatalyst is initiated by free radicals formed by the reaction of light irradiation. When the irradiated light energy is increased higher than bandgap energy of catalytic materials, electrons in valence band (VB) are excited to conduction band (CB), leaving holes in the valence band, resulting in electron–hole pairs on the surface of the catalyst [52]. This excited surface with hole-electron pairs is more active in redox reactions with molecules adsorbed on the surface. The holes formed can act as reducing agents. The photocatalytic mechanism of PMMA-wrapped TiO_2_ for degradation of methylene blue dye is illustrated in Figure S2. The light irradiated on TiO_2_/PMMA film liberates holes and electrons from the composite, and the hole can further produce hydroxyl radicals from the organic dye molecules through oxidation. The electrons can also react with the dye molecules and produce reduction products. In a typical process, the valence band holes are used to form hydroxyl radicals by reacting with water and hydroxyl ions, whereas the generated electrons reduce the adsorbed oxygen molecules as superoxide radical anions, which again forms peroxide radicals by the action of holes, and, finally, dye is degraded.

## 4. Conclusions

Nanoparticle-incorporated PMMA polymer nanocomposites were successfully prepared using a simple solution method. CNT/PMMA, TiO_2_/PMMA, and CQD/PMMA composites were created as freestanding films suitable for various high-tech applications. The prepared films’ structural, morphological, and optical properties were characterized, and their optical parameters were investigated, including refractive index and dielectric constants. The CNT/PMMA and TiO_2_/PMMA films exhibited lower bandgaps than their individual parts, which is attractive for new and innovative applications. The CNT/PMMA film exhibited good electrical conductivity and would be a suitable flexible substrate for other applications. The TiO_2_/PMMA film exhibited efficient photocatalytic degradation of methylene blue dye under sunlight, which could be useful for wastewater treatment, providing self-cleaning antimicrobial and UV shielding layers. The CQD/PMMA film exhibited good fluorescence effects across the visible range and would be useful for antireflective coatings. Hence, this work proposes a facile method to prepare novel and application-selective nanoparticle and polymer composite freestanding films.

## Data Availability

The data presented in this study are available on request from the corresponding due to ethical reasons.

## Supplementary Materials

The following supporting information can be downloaded at: https://www.mdpi.com/article/10.3390/polym16081048/s1, Figure S1: Absorption spectrum of methylene blue dye at different UV exposure with TiO_2_ nanoparticles. Figure S2. Schematic representation of photocatalytic degradation mechanism of TiO_2_/PMMA on MB dye. Table S1: Comparison of MB dye degradation efficiency of TiO_2_/PMMA films with literature values. References [53,54,55,56,57,58,59] are cited in the Supplementary Materials.
