# Supplementary material for: Optical and UV Shielding Properties of Inorganic Nanoparticles Embedded in Polymethyl Methacrylate Nanocomposite Freestanding Films
Source: Polymers (Basel). 2024 Apr 10;16(8):1048. doi: 10.3390/polym16081048 (PMC11053758; doi:10.3390/polym16081048)
Supplement: Supplementary file 1 [file polymers-16-01048-s001.zip › polymers-2911461-supplementary.pdf]

Optical and UV shielding properties of inorganic nanoparticles embedded PMMA nanocomposite freestanding films

A. Kathalingam[a], Dhanasekaran Vikraman[b], Du-Hee Lee[b], Yoon-A Cho[b], Hyun-Seok Kim[b*]

[a]*Millimeter-wave Innovation Technology Research Center, Dongguk University-Seoul, Seoul 04620, Republic of Korea*

[b]*Division of Electronics and Electrical Engineering, Dongguk University-Seoul, Seoul 04620, Republic of Korea*

Corresponding author. E-mail address:* hyunseokk@dongguk.edu

Abstract

Polymethyl methacrylate (PMMA) is an interesting polymer employed in various applications due its outstanding properties. However, its electrical and mechanical properties can be further improved by incorporating nanoparticles, and in particular PMMA nanocomposite with nanoparticles provide various multifunctional properties. This work reports PMMA nanocomposite preparation and structural and optical characterizations incorporating carbon nanotubes (CNT), TiO_2 nanoparticles and carbon quantum dots (CQD). CNT/PMMA, TiO_2/PMMA, and CQD/PMMA nanocomposite freestanding films were prepared using a simple solution method. Various properties for the prepared composite films are reported using scanning electron microscopy, X-ray diffraction, photoluminescence, Fourier transform infrared, and UV-Vis spectroscopy. Optical parameters and photocatalytic dye degradation for the films are reported focusing on materials properties. The CNT/PMMA, TiO_2/PMMA, and CQD/PMMA films

achieved respectively good electrical conductivity, photodegradation, and fluorescence, compared with other composite films.

Figure S1. Absorption spectrum of methylene blue dye at different UV exposure with TiO_2 nanoparticles

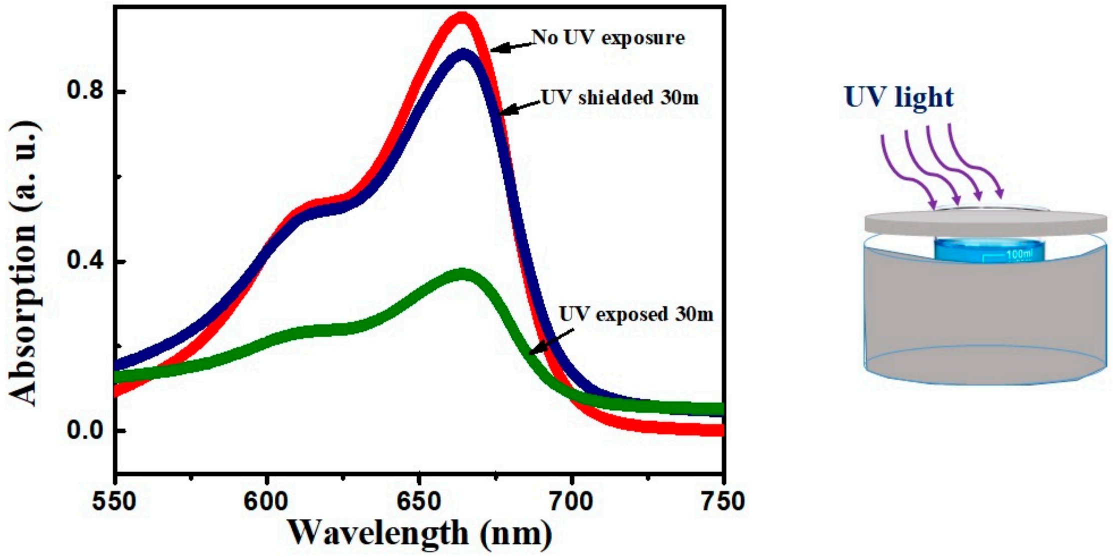

Figure S2. Schematic representation of photocatalytic degradation mechanism of TiO_2/PMMA on MB dye

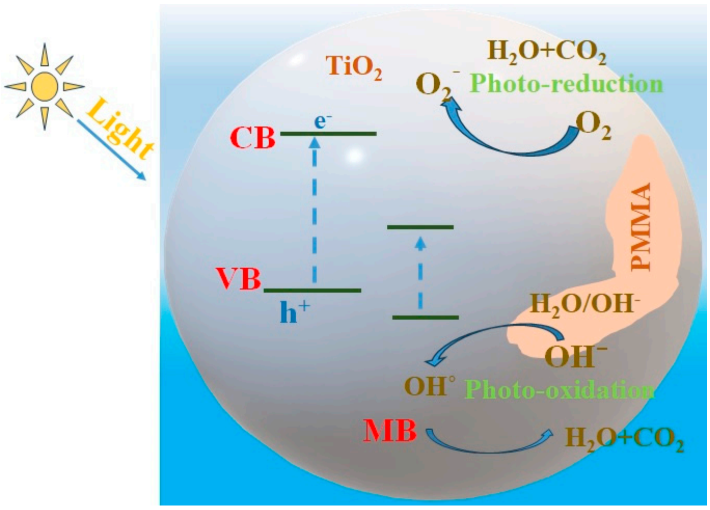

Table S1. Comparison of MB dye degradation efficiency of TiO_2/PMMA films with literature values

Materials	Radiation type	Catalyst concentration	Time (min)	Efficiency %	Ref
Fe_2TiO_5	Solar light	50 mg	240	55	[1]
NDCQDs/ZIF-8	Solar light	-	180	93	[2]
(CeO_2-NPs)/GO/PAM	UV-light	-	90	90	[3]
$CoZn_5MgFeO_4$	Solar light	-	-	99	[4]
rGO@ZnO	Solar light	20 mg	-	99	[5]
$MgFe_2O_4$	Solar light	-	35	75	[6]
MO-PMMA	Solar light	-	400	99	[7]
TiO_2/PMMA	Solar light	Not applicable Freestanding film	60	40	Present work

References

1. Vasiljevic, Z.; Dojcinovic, M.; Vujancevic, J.; Jankovic-Castvan, I.; Ognjanovic, M.; Tadic, N.; Stojadinovic, S.; Brankovic, G.; Nikolic, M. Photocatalytic degradation of methylene blue under natural sunlight using iron titanate nanoparticles prepared by a modified sol–gel method. *Royal Society open science* **2020**, *7*, 200708.
2. Abd El Khalk, A.A.; Betiha, M.A.; Mansour, A.S.; Abd El Wahed, M.G.; Al-Sabagh, A.M. High degradation of methylene blue using a new nanocomposite based on zeolitic imidazolate framework-8. *ACS omega* **2021**, *6*, 26210-26220.
3. Kalaycıoğlu, Z.; Uysal, B.; Pekcan, Ö.; Erim, F. Efficient photocatalytic degradation of methylene blue dye from aqueous solution with cerium oxide nanoparticles and graphene oxide-doped polyacrylamide. ACS Omega 8: 13004–13015. **2023**.
4. Chahar, D.; Kumar, D.; Thakur, P.; Thakur, A. Visible light induced photocatalytic degradation of methylene blue dye by using Mg doped Co-Zn nanoferrites. *Materials Research Bulletin* **2023**, *162*, 112205.
5. Negash, A.; Mohammed, S.; Weldekirstos, H.D.; Ambaye, A.D.; Gashu, M. Enhanced photocatalytic degradation of methylene blue dye using eco-friendly synthesized rGO@ ZnO nanocomposites. *Scientific Reports* **2023**, *13*, 22234.
6. Cabrera, A.; Torres, C.R.; Marchetti, S.; Stewart, S. Degradation of methylene blue dye under dark and visible light conditions in presence of hybrid composites of nanostructured MgFe2O4 ferrites and oxygenated organic compounds. *Journal of Environmental Chemical Engineering* **2020**, *8*, 104274.
7. Rani, M.; Shanker, U. Sun-light driven rapid photocatalytic degradation of methylene blue by poly (methyl methacrylate)/metal oxide nanocomposites. *Colloids and Surfaces A: Physicochemical and Engineering Aspects* **2018**, *559*, 136-147.
